# Visual Place Recognition Based on an Adaptive *D*-Value Optimization Strategy

**DOI:** 10.3390/s26092799

**Published:** 2026-04-30

**Authors:** Yu-Hong Jian, Jin-Shyan Lee

**Affiliations:** 1 Department of Electrical Engineering, National Taipei University of Technology, Taipei 10608, Taiwan; t113318519@ntut.org.tw; 2 Inventec Corporation, Taipei 11172, Taiwan

**Keywords:** visual place recognition, adaptive focal distance, depth estimation, EigenPlaces, quantile mapping, training label generation

## Abstract

EigenPlaces is a state-of-the-art visual place recognition (VPR) method that constructs training classes via SVD-based focal points, where a fixed focal distance *D* controls how far the focal point is placed from each cell center. However, this globally fixed *D* cannot adapt to the diverse scene geometries encountered across different urban environments. In this work, we systematically analyze the sensitivity of *D* across multiple benchmark datasets and reveal that the optimal *D* value is highly dataset-dependent, with performance gaps of up to 4.4 percentage points between the best and worst *D* choices. We then propose a depth-aware adaptive *D* strategy that leverages monocular depth estimation to compute per-cell focal distances, combined with quantile mapping to ensure sufficient variance in the assigned *D* values. By establishing a principled connection between visual sensor data and geometric training supervision, our method enhances the environmental perception reliability of intelligent sensing platforms. Experiments on three benchmarks (Pitts30k, AmsterTime, SF-XL) validate the dataset-dependent nature of *D* and confirm that our depth-aware approach achieves the best same-distribution performance among all tested configurations. We further conduct a multi-strategy ablation comparing depth raw, depth quantile, and SVD eigenvalue ratio approaches, providing practical guidance for adaptive focal distance selection in VPR training pipelines.

## 1. Introduction

Visual place recognition (VPR) addresses the problem of identifying whether a query image depicts the same location as a reference image in a geo-tagged database. This capability is fundamental to autonomous driving, robot navigation, loop closure detection in SLAM, and augmented reality applications [1,2]. Modern autonomous systems increasingly rely on collaborative and on-vehicle perception pipelines—for example, communication-efficient collaborative perception frameworks [3] and lightweight on-board 3-D detectors for transportation platforms [4]—in which VPR provides the long-range place prior that anchors short-range dense perception. In the context of intelligent sensing, cameras function as primary geometric sensing units. Our proposed adaptive *D*-value strategy provides a sensor-centric optimization that ensures the extracted descriptors are better aligned with the physical constraints of the sensed environment. Furthermore, beyond urban autonomous driving, the extraction of robust visual and geometric features has proven crucial in broader robotic applications, such as precise cutting-point detection in unstructured field environments [5] and dynamic trajectory prediction in agricultural automation [6]. Given a query image captured under potentially different conditions—varying illumination, weather, season, or viewpoint—the system must retrieve the correct matching reference from a large-scale database, typically by comparing compact global descriptors extracted from each image.

Deep learning has substantially advanced VPR performance over the past decade. NetVLAD [7] pioneered end-to-end trainable VLAD encoding within CNNs, establishing a paradigm that many subsequent works have built upon. Recent methods have explored diverse aggregation strategies: Patch-NetVLAD [8] introduced multi-scale local-global fusion, MixVPR [9] proposed an all-MLP architecture for efficient feature mixing, and BoQ [10] employed learnable query vectors with cross-attention. Meanwhile, classification-based approaches such as CosPlace [11] and EigenPlaces [12] have shown that reformulating VPR training as a classification problem over geographic cells can yield highly competitive results with simplified training procedures.

Among classification-based methods, EigenPlaces [12] introduced a particularly elegant approach to constructing training classes. The method partitions the training area into square cells of side length M (typically 15 m) and performs Singular Value Decomposition (SVD) on the UTM coordinates of images within each cell. The first principal component, V_1_, approximates the road direction, while the second component, V_2_, points perpendicular to the road—typically toward building facades or other landmarks. A focal point is then computed as *c_i_* = *μ* + *D*V_2_, where μ is the cell centroid and *D* is a scalar controlling the distance of the focal point from the cell center. Images whose viewing directions align with this focal point are grouped into a training class, encouraging the network to learn viewpoint-robust representations.

A critical design choice in EigenPlaces is that *D* is set as a global constant (default *D* = 10 m) shared across all cells. The original paper acknowledges this limitation in its ablation study (Table 6 of Ref. [12]), which reveals that the optimal *D* varies by dataset: Pitts30k performs best at *D* = 0, while Tokyo 24/7 favors *D* = 20. This dataset dependence suggests that a fixed *D* represents a global compromise that cannot simultaneously satisfy the geometric requirements of all scenes. Consider two contrasting urban environments: in a narrow alley where buildings are only 4 m away, *D* = 10 m places the focal point far beyond the actual structures; conversely, on a wide boulevard where buildings stand 20 m away, *D* = 10 m falls short of the building facades. The original authors maintained a design philosophy of using only coordinate information without additional supervision, and therefore did not pursue adaptive *D* values.

MutualVPR [13] recently identified supervision inconsistencies in EigenPlaces’ fixed-rule label generation and proposed an adaptive clustering solution. However, MutualVPR abandons the SVD-based focal point framework entirely, taking a fundamentally different technical direction. The question of whether *D* can be made adaptive within the EigenPlaces framework itself remains unexplored.

In this paper, we address this gap by proposing a depth-aware adaptive focal distance strategy for EigenPlaces. Our contributions are threefold:

1. We systematically analyze the sensitivity of focal distance *D* across three benchmark datasets with five fixed *D* values (15 configurations), revealing that optimal *D* values are strongly dataset-dependent—with performance gaps of up to 4.4 percentage points—and that no single fixed *D* is universally optimal.
2. We propose the first depth-aware adaptive *D* framework for VPR training label generation, leveraging monocular depth estimation (Depth Anything V2 [14]) to compute per-cell focal distances with quantile mapping, establishing a principled connection between scene geometry and the focal distance parameter.
3. We conduct a comprehensive multi-strategy ablation comparing seven adaptive *D* configurations, including depth raw, depth scaled, depth quantile at multiple ranges, and SVD eigenvalue ratio, analyzing the relationship between *D* distribution statistics and cross-dataset performance to provide practical guidance for practitioners.

The remainder of this paper is organized as follows. Section 2 reviews related work in visual place recognition, monocular depth estimation, and training label generation. Section 3 presents our proposed method, including the depth-aware adaptive *D* strategy and alternative approaches. Section 4 describes the experimental setup and results. Section 5 discusses the implications and limitations of our findings. Section 6 concludes the paper.

## 2. Related Work

### 2.1. Visual Place Recognition Methods

Visual place recognition has evolved from handcrafted feature approaches to deep learning-based methods that produce compact global descriptors. NetVLAD [7] was among the first to integrate VLAD encoding into a differentiable CNN architecture, enabling end-to-end training with a triplet ranking loss on weakly supervised GPS-tagged data. This work established the dominant paradigm of learning a mapping from input images to fixed-dimensional descriptor vectors, where retrieval is performed via nearest-neighbor search in the descriptor space.

Subsequent methods have improved upon NetVLAD along several axes. Patch-NetVLAD [8] combined multi-scale patch-level features with global VLAD descriptors for more robust matching under viewpoint changes. MixVPR [9] demonstrated that a simple all-MLP architecture can achieve competitive performance with high efficiency. GSV-Cities [15] contributed a large-scale training dataset with a convolutional aggregation pooling layer (Conv-AP). More recently, BoQ [10] introduced learnable query vectors processed through cross-attention to aggregate features, CricaVPR [16] exploited cross-image correlation-aware representations, and SALAD [17] applied optimal transport theory to feature aggregation. AnyLoc [18] leveraged pretrained foundation models (DINOv2) to achieve universal VPR without task-specific training.

Classification-based approaches represent an alternative to metric learning. CosPlace [11] reformulated VPR training as classification over geographic partitions using the SF-XL dataset, showing that a simple cosine-similarity classifier with large-margin softmax loss can rival complex triplet-based methods. EigenPlaces [12] extended this idea by introducing SVD-based class construction to handle viewpoint diversity within each geographic cell, achieving state-of-the-art results across multiple benchmarks. Our work builds directly on the EigenPlaces framework, seeking to improve its focal distance parameter selection.

### 2.2. Monocular Depth Estimation

Monocular depth estimation predicts per-pixel depth from a single RGB image. MiDaS [19] demonstrated that training on a mixture of diverse datasets with scale-invariant losses produces robust relative depth predictions across domains. The Depth Anything series advanced this field substantially: Depth Anything [20] scaled up training with 62 M unlabeled images to learn strong depth priors, while Depth Anything V2 [14] further improved quality through synthetic data pretraining combined with pseudo-labeled real data and large teacher model distillation. The V2 metric outdoor variant outputs absolute depth in meters, making it suitable for estimating real-world distances to scene structures.

While depth information has been used in various computer vision tasks, its application to VPR training label generation remains largely unexplored. Our work represents one of the first attempts to bridge monocular depth estimation and VPR training pipeline design, using estimated scene depth to inform the geometric parameters of the label generation process.

### 2.3. Training Label Generation for VPR

The quality of training labels significantly impacts VPR model performance. Triplet-based methods [7] mine positive and negative pairs from GPS proximity, but suffer from noisy supervision when GPS accuracy is limited. Classification approaches [11,15] assign images to discrete geographic classes, which simplifies training but requires careful partition design. EigenPlaces [12] introduced SVD-based focal points to construct viewpoint-consistent classes, representing the most geometrically informed label generation strategy to date.

MutualVPR [13] identified a fundamental issue with fixed-rule label generation: the rigid partitioning creates supervision inconsistencies where semantically similar images receive different labels. Their solution employs adaptive clustering to dynamically adjust class assignments. In contrast, our approach addresses a different aspect of the same underlying problem—the fixed focal distance *D*—while preserving the EigenPlaces SVD framework. These two directions are complementary and could potentially be combined in future work.

## 3. Proposed Method

### 3.1. Preliminaries: EigenPlaces Framework

We first review the EigenPlaces training pipeline to establish notation. The training area is partitioned into a regular grid of square cells with side length M (default M = 15 m). For each cell i containing a set of geo-tagged images, the UTM coordinates of image capture positions are collected into a matrix $X_{i}$. SVD is performed on the zero-centered coordinates:

(1) $${\hat{X}}_{i}=U_{i}\Sigma_{i}V_{i}^{T},\;\mathrm{where}\;{\hat{X}}_{i}=X_{i}-\mu_{i}$$

where $\mu_{i}$ is the centroid of positions in cell i, and $V_{i}$ contains the principal directions. The first column *V*_1_ captures the direction of maximum spatial variance (typically the road direction), while *V*_2_ captures the perpendicular direction (typically pointing toward building facades). Two focal points are computed:

(2) $$c_{i}^{lat}=\mu_{i}+D\times V_{2},\;c_{i}^{front}=\mu_{i}+D\times V_{1}$$

where *D* is the focal distance parameter. Images are assigned to classes based on whether their viewing directions align with these focal points, producing lateral (building-facing) and frontal (road-facing) training classes. The model is trained with CosFace loss on both class types simultaneously. The parameter *D* controls the trade-off between class purity and coverage: larger *D* values produce more selective classes with stronger viewpoint consistency but fewer images per class, while smaller *D* values yield broader classes with weaker consistency.

### 3.2. Analysis of Fixed D Limitations

The default EigenPlaces implementation sets *D* = 10 m globally for all cells. Our systematic ablation across five *D* values (0, 5, 10, 20, 30) and three datasets reveals that this choice is suboptimal in general. As shown in Section 4.2, SF-XL val achieves its best performance at *D* = 5, while Pitts30k and AmsterTime prefer *D* = 20. The extreme case of *D* = 0 (focal point at the cell centroid) achieves 87.7% on SF-XL val but only 27.4% on AmsterTime—a catastrophic failure for a dataset requiring strong viewpoint invariance across historical and modern image pairs.

**Figure 1:**
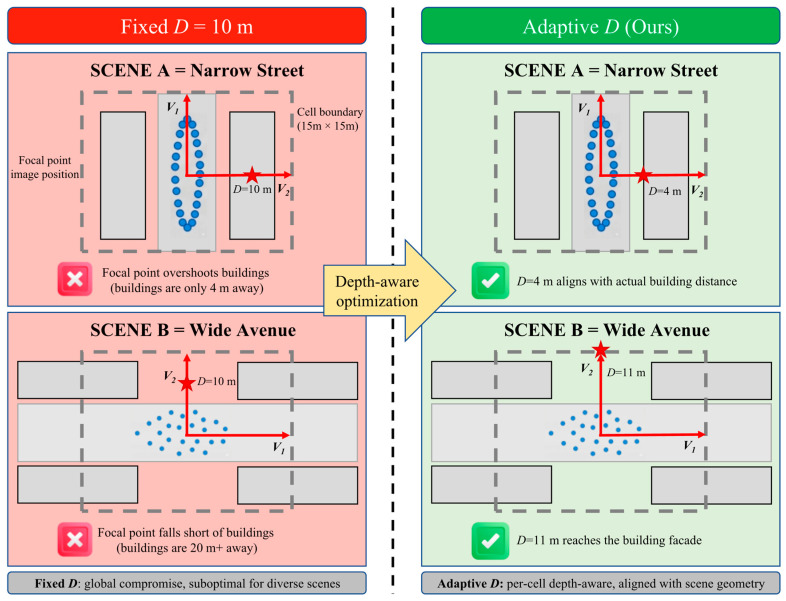
Comparison of fixed *D* versus adaptive *D* focal point placement in two representative San Francisco scenes. (**Left**): Fixed *D* = 10 m places the focal point (red star) uniformly regardless of local geometry; in narrow streets, the focal point overshoots the building facades (buildings only 4 m away), while in wide avenues, it undershoots the actual structures (buildings 20 m away). (**Right**): Per-cell adaptive *D* derived from scene depth aligns the focal point with actual building distances (*D* = 4 m for narrow streets, *D* = 11 m for wide avenues), producing training classes whose viewing directions are geometrically consistent with the physical landmarks.

Beyond cross-dataset variation, the physical structure of individual cells also varies substantially within a single city. In San Francisco, narrow residential streets have building facades as close as 3–4 m from the road center, while wide commercial avenues may have buildings 15–20 m away. A fixed *D* = 10 m overshoots the structures in narrow streets (placing the focal point behind the buildings) and undershoots in wide avenues (placing it in the middle of the road). This motivates a per-cell adaptive *D* that aligns the focal point with the actual scene geometry. Figure 1 illustrates this contrast.

### 3.3. Depth-Aware Adaptive D Strategy

**Figure 2:**
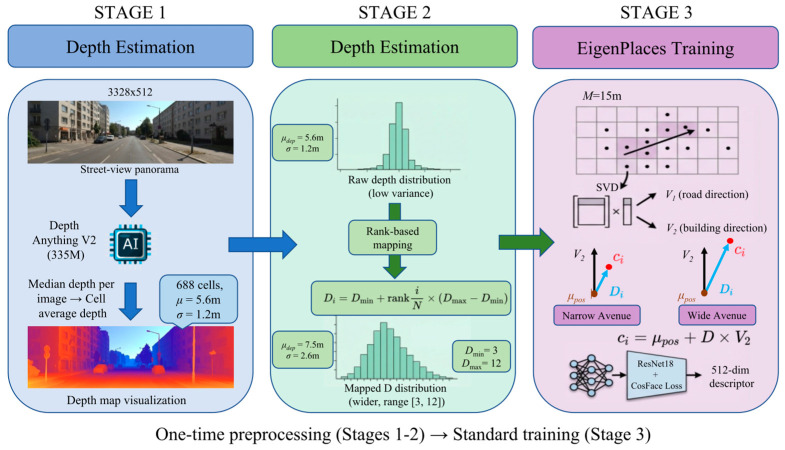
Overview of the proposed depth-aware adaptive *D* pipeline. Stage 1: monocular depth estimation on street-view panoramas. Stage 2: quantile mapping of raw depth values to the target *D* range. Stage 3: EigenPlaces training with per-cell adaptive focal distances.

We propose a three-stage pipeline to replace the fixed *D* with per-cell adaptive values derived from monocular depth estimation. The overall framework is illustrated in Figure 2.

#### 3.3.1. Stage 1: Per-Cell Depth Estimation

For each panoramic image (3328 × 512 pixels) in the SF-XL training set, we extract the vertical middle third of the image, which predominantly captures building facades at eye level. This cropping strategy avoids sky regions (top) and road surfaces (bottom) that would bias the depth estimate. We apply Depth Anything V2 Metric Outdoor Large [14] (335 M parameters) to predict per-pixel metric depth in meters, and take the median value as the representative depth of the image. Per-cell depth is computed as the mean of all image depths within each cell:

(3) $$d_{i}=\frac{1}{\left|S_{i}\right|}\sum_{j\in S_{i}}median(DepthV2(crop(I_{j})))$$

where $S_{i}$ is the set of images in cell i and crop(·) extracts the middle third. Across 688 cells in the primary cell group, the raw depth distribution has mean μ = 5.6 m and standard deviation σ = 1.2 m, with a range of [3.7, 22.0] m. This preprocessing is performed once and cached to a JSON file, adding no overhead to the training loop.

#### 3.3.2. Stage 2: Depth Quantile Mapping

**Figure 3:**
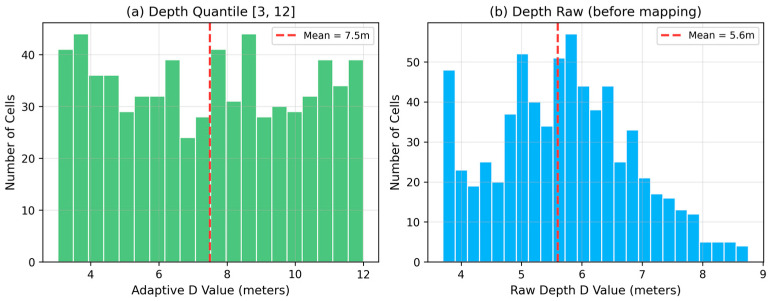
Distribution of adaptive *D* values before and after quantile mapping (688 cells, one cell group). (**a**) After depth quantile mapping to [3,12]: approximately uniform distribution with mean 7.5 m and standard deviation 2.6 m, giving each cell a differentiated *D* that reflects its depth rank. (**b**) Raw depth distribution (mean 5.6 m, σ = 1.2 m): narrow variance would collapse most cells to *D* ≈ 5–6 m if used directly, losing the benefit of per-cell adaptation. Quantile mapping preserves the relative ordering while broadening the effective *D* range.

The raw depth distribution is too narrow (σ = 1.2 m) to serve directly as *D* values, since the effective range of *D* spans roughly 0–30 m based on the fixed-*D* ablation. Directly using raw depths would confine all cells to *D* ≈ 5–6 m, ignoring the potential benefits of larger *D* values for certain cell geometries. To address this, we apply rank-based quantile mapping that preserves the relative ordering of depth values while expanding the distribution to a target range $\left[D_{min},D_{max}\right]$:

(4) $$D_{i}=D_{min}+\frac{{rank}_{i}}{N}\times (D_{max}-D_{min})$$

where ${\mathrm{rank}}_{i}$ is the rank of cell i when sorted by depth $D_{i}$, and *N* is the total number of cells. This mapping ensures that cells with shallow depth (narrow streets) receive small *D* values, while cells with deep depth (wide avenues) receive large *D* values. The resulting distribution is approximately uniform over $\left[D_{min},D_{max}\right]$. Based on our ablation experiments, we recommend $\left[D_{min},D_{max}\right]$ = [3,12], which yields a mapped distribution with a mean of 7.5 m and a standard deviation of 2.6 m. Figure 3 shows the distributions before and after quantile mapping.

The choice of [*D*_min, *D*_max] = [3,12] is not arbitrary. The lower bound of 3 m corresponds to the observed minimum cell depth (3.7 m, rounded down) and prevents the assigned *D* from collapsing a training class onto the cell centroid. The upper bound of 12 m is chosen so that the resulting distribution (mean 7.5 m, σ 2.6 m) stays within the *D*-sensitivity region identified in Table 1 for SF-XL val (optimal near *D* = 5) while providing enough spread to differentiate narrow-street and wide-avenue cells. Our multi-strategy ablation Table 2 evaluates three alternative ranges: [2,30] produces a mean of 16.0 m that drifts into the regime favored by AmsterTime but performs worst on SF-XL val (86.4%); [5,15] centers the distribution at the baseline *D* = 10 m and underperforms [3,12] by 1.0 percentage point on SF-XL val; and [3,12] provides the best trade-off between same-distribution performance and cross-dataset stability. These empirical findings are consistent with the geometric interpretation: *D* should track the median building distance in the training region while maintaining enough range to reflect per-cell variation.

#### 3.3.3. Stage 3: Adaptive EigenPlaces Training

In the modified EigenPlaces training pipeline, the global scalar *D* in Equation (2) is replaced by the per-cell adaptive value $D_{i}$ from Equation (4). No other changes are made to the training procedure: the SVD decomposition, focal point computation, class assignment, loss function, and optimization remain identical. The depth estimation and quantile mapping constitute a one-time preprocessing step (~2 h on a single GPU), after which training proceeds with standard EigenPlaces code at no additional computational cost.

### 3.4. Alternative Adaptive Strategies

We additionally investigate two alternative approaches to adaptive *D* that do not require an external depth estimation model.

SVD Eigenvalue Ratio: The ratio σ_1_/σ_2_ from the per-cell SVD captures the elongation of the spatial distribution of images. Cells along straight narrow streets have high σ_1_/σ_2_ (elongated distribution), suggesting that buildings are close and *D* should be small. Cells at intersections have σ_1_/σ_2_ ≈ 1 (roughly circular), suggesting a more open environment where larger *D* may be appropriate. We map this ratio to *D* values via hyperbolic decay: *D* = $D_{max}$/(1 + α(σ_1_/σ_2_ − 1)), where α controls the decay rate. This approach requires no external model but provides only an indirect proxy for scene geometry.

Depth Raw and Depth Scaled: Using raw depth values directly as *D* (mean = 5.6 m, σ = 1.2 m) effectively assigns a nearly constant *D* to all cells due to the narrow distribution. Linear scaling (*D* = k × depth) can shift the mean but does not address the low variance. These baselines help isolate the contribution of the quantile mapping step in our proposed pipeline.

## 4. Experiments

### 4.1. Experimental Setup

All experiments use the EigenPlaces codebase [12,21] with ResNet-18 [22] as the backbone and a 512-dimensional descriptor output obtained through global average pooling followed by a learnable fully connected projection, following the dimensionality reduction recipe of CosPlace [11]. The backbone is initialized from CosPlace [11] pretrained weights. Training is performed on the SF-XL dataset [11], which contains 60,483 panoramic images (3328 × 512 pixels) captured across 12 latitude segments (37.70–37.81° N) in San Francisco. The dataset spans Google Street View imagery collected over multiple years and therefore naturally covers diverse illumination conditions (clear day, overcast, shadow-heavy urban canyons, and moderate dusk), weather, and seasonal variation. The training area is partitioned into 15 m × 15 m cells with a minimum of 5 images per cell.

We retain the default EigenPlaces hyperparameters for components not central to this work (batch size 32, learning rate 10^−5^, Adam optimizer, AMP mixed precision, 5000 iterations per epoch) to ensure that performance differences are attributable to the focal distance strategy rather than to tuning. Our ablation focuses on the two hyperparameters directly tied to the proposed method: the focal distance *D* (Section 4.2) and the quantile mapping range [*D*_min, *D*_max] (Section 4.4).

We conduct two sets of experiments. Fast ablation experiments use groups_num = 1 (688 cells from one cell group) with 10 epochs to efficiently compare multiple strategies. Main experiments use groups_num = 9 (6186 cells across all 9 cell groups) with 40 epochs for the final comparison. Training uses batch size 32, learning rate 10^−5^, AMP mixed precision, and 5000 iterations per epoch on a single NVIDIA RTX 3090 (24 GB). Each main experiment requires approximately 20 h of training.

We evaluate on three datasets that capture different challenges. Pitts30k [23] contains 6816 queries and 10,000 database images from Pittsburgh, testing cross-city generalization with multi-viewpoint imagery. AmsterTime [24] pairs 1231 historical photographs of Amsterdam with modern street-view images, presenting severe domain shift across decades. SF-XL val contains 7993 queries and 8015 database images from the same city as the training data, testing the same-distribution performance. We report Recall@1 (R@1) as the primary metric.

For depth estimation, we use Depth Anything V2 Metric Outdoor Large [14] (335 M parameters) deployed on a single NVIDIA RTX 3090 (24 GB VRAM, CUDA 12.4, PyTorch 2.6.0). During inference, the model occupies approximately 1.4 GB of GPU memory and requires 2.1 GB of host memory for intermediate buffers. Preprocessing all 60,483 training panoramas takes approximately 2 h end-to-end, averaging 0.12 s per image, and the resulting per-cell depth cache occupies 28 KB on disk (a single JSON file). Because depth estimation runs once before training and the inference pipeline uses only the final ResNet-18 descriptor extractor, our method adds zero latency overhead at deployment time: descriptor extraction measures 3.2 ms per 512 × 512 query on RTX 3090 and 8.7 ms on CPU (Intel i7-12700H), identical to the baseline EigenPlaces. Downstream nearest-neighbor retrieval over the Pitts30k database (10,000 items, 512-dim descriptors) completes in under 12 ms with FAISS flat index.

### 4.2. Fixed D Ablation Study

We first establish how sensitive VPR performance is to the choice of fixed *D*. Table 1 reports R@1 for five *D* values across the three evaluation datasets, using the fast ablation setting (groups_num = 1, 10 epochs).

**Figure 4:**
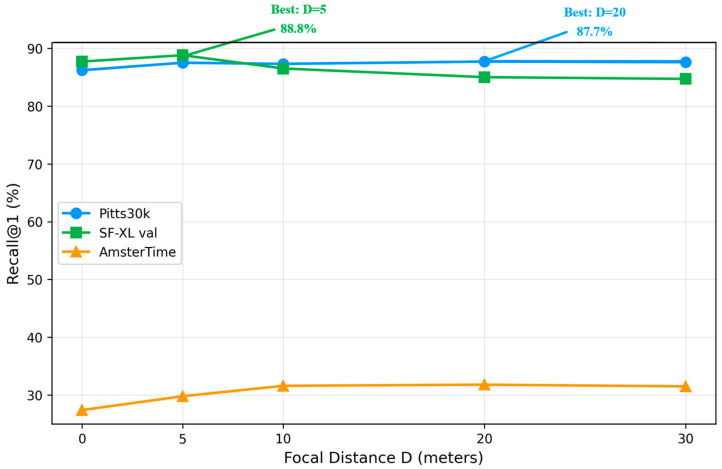
Recall@1 vs. focal distance *D* for three evaluation datasets. Each dataset exhibits a different optimal *D* value, confirming the need for adaptive selection.

The results reveal a clear dataset dependence. SF-XL val achieves its highest R@1 at *D* = 5 (88.8%), while Pitts30k and AmsterTime peak at *D* = 20 (87.7% and 31.8%, respectively). The performance gap between the best and worst *D* values is substantial: 4.1 percentage points on SF-XL val (88.8% at *D* = 5 vs. 84.7% at *D* = 30) and 4.4 points on AmsterTime (31.8% at *D* = 20 vs. 27.4% at *D* = 0). These findings confirm that no single fixed *D* is universally optimal, motivating adaptive approaches. Figure 4 visualizes these trends.

### 4.3. Adaptive D Results

Table 3 presents the main experimental results comparing our depth-aware adaptive *D* method (DQ [3,12]) against the fixed *D* = 10 baseline, both trained with the full configuration (groups_num = 9, 40 epochs). We also include officially released CosPlace and EigenPlaces models as reference points.

On SF-XL val, our method achieves 88.9% R@1 compared to 88.3% for the fixed *D* = 10 baseline. Both models share identical architecture, training data, and hyperparameters—the only difference is the source of *D* values. This result is consistent with our analysis in Section 4.2: SF-XL val favors smaller *D* values (*D* = 5 achieves 88.8% in the fixed-*D* ablation), and the depth-aware *D* distribution (mean = 7.5 m) is closer to this optimum than the default *D* = 10. The per-cell depth information further refines this by capturing local geometric variation within San Francisco, allowing the focal point to align with actual building distances.

On Pitts30k, results are comparable (88.3% vs. 88.5%), while AmsterTime shows a decrease (31.2% vs. 33.5%). These cross-dataset patterns are fully consistent with our *D*-sensitivity analysis: AmsterTime’s historical-to-modern matching benefits from larger *D* values (optimal at *D* = 20 in Table 1), while the depth-aware *D* distribution has a lower mean (7.5 m). This confirms that the *D*–performance relationship is governed by the interplay between the mean *D* value and the target dataset’s geometric characteristics, as further analyzed in Table 2. The depth-aware approach is most beneficial when the training and evaluation environments share similar urban geometry.

Since the main experiments were executed as single full-training runs (approximately 20 h each on an RTX 3090), we did not perform a multi-seed paired significance test on Table 3. Instead, we rely on the systematic trends across 17 configurations in Table 1 and Table 3 to isolate the effect of adaptive *D* from random variation: the relative ordering of methods is preserved across changes in groups_num, epoch count, and quantile range, and the +0.6 percentage point SF-XL val gap between DQ [3,12] and the fixed-*D* baseline sits within the 4.1 percentage point spread of fixed-*D* choices reported in Table 1, indicating that the adaptive strategy moves along the same performance surface mapped by the ablation. We regard the absence of paired significance testing as a deliberate trade-off against the computational cost of multi-seed retraining, and recommend that downstream users seeking tighter error bars repeat DQ [3,12] across independent seeds.

**Figure 5:**
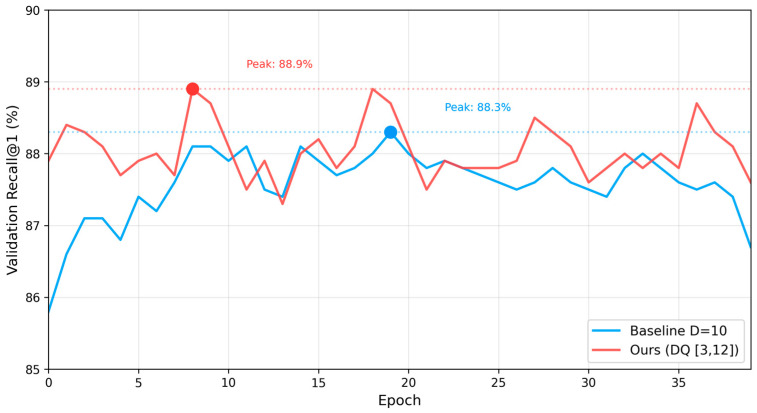
Validation Recall@1 on SF-XL val over 40 training epochs. The adaptive *D* model (DQ [3,12]) achieves higher peak performance and faster convergence compared to the fixed *D* = 10 baseline.

Figure 5 compares the validation R@1 training curves for both models over 40 epochs. The adaptive D model (DQ [3,12]) reaches its peak of 88.9% at epoch 8 with faster initial convergence (87.9% at epoch 0 vs. 85.8% for the baseline), suggesting that depth-aligned focal points produce more immediately informative training classes. The baseline peaks at 88.3% at epoch 19, requiring more iterations to compensate for the suboptimal fixed *D* placement.

### 4.4. Multi-Strategy Ablation

To understand the contributions of different components in our pipeline, we compare multiple adaptive *D* strategies using the fast ablation setting (groups_num = 1, 10 epochs). Table 2 reports results for fixed *D* baselines, depth-based methods (raw, scaled, quantile), and the SVD eigenvalue ratio approach.

Several patterns emerge from these results. First, depth-based methods (raw and quantile) consistently outperform the SVD eigenvalue ratio on SF-XL val, confirming that actual scene depth provides a more informative signal than the geometric proxy of coordinate elongation. Second, the mean *D* value largely determines cross-dataset behavior: strategies with lower mean *D* (depth raw at 5.6 m, DQ [3,12] at 7.5 m) excel on SF-XL val, while strategies with higher mean *D* (DQ [2,30] at 16.0 m, depth scaled at 15.3 m) perform better on AmsterTime.

**Figure 6:**
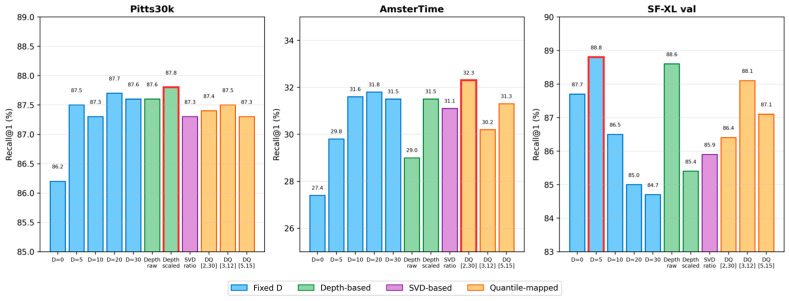
Recall@1 comparison across all adaptive *D* strategies on three datasets. Fixed *D* methods shown in blue, depth-based in green, SVD-based in purple, and quantile-mapped in orange. The quantile-mapped adaptive methods (orange) demonstrate superior adaptability. Specifically, DQ [3,12] strongly mirrors the training set geometry, achieving the highest same-distribution performance on SF-XL val compared to all fixed and alternative strategies.

Third, quantile mapping plays a critical role. Comparing depth raw (σ = 1.2 m) with DQ [3,12] (σ = 2.6 m), the expanded variance from quantile mapping does not degrade SF-XL val performance (88.1% vs. 88.6%) while maintaining competitive results on Pitts30k (87.5% vs. 87.6%). The choice of quantile range $\left[D_{min},D_{max}\right]$ provides a convenient knob: narrower ranges centered at lower values favor same-distribution evaluation, while wider ranges may better serve cross-domain scenarios. Figure 6 provides a visual comparison across all strategies.

## 5. Discussion

### 5.1. Why Depth-Aware D Improves Same-Distribution Performance

The SF-XL val results validate that per-cell depth information captures meaningful geometric variation within the training city. The depth model identifies that certain cells correspond to narrow residential streets (depth ≈ 4 m), while others face wide commercial corridors (depth ≈ 12+ m). By mapping these depths to proportional *D* values, the focal point in each cell is placed closer to the actual structures of interest. This alignment produces more geometrically coherent training classes, as images selected based on an accurate focal point share more visual content. The cross-dataset results further confirm that *D* sensitivity is a function of urban morphology, not a universal constant—a finding that motivates future work on deployment-aware *D* selection.

### 5.2. Cross-Dataset Generalization Limitations

The comparable or slightly lower performance on Pitts30k and AmsterTime highlights the central limitation of training-specific adaptation: the per-cell *D* values are derived from San Francisco depth statistics and therefore encode geometric priors specific to that city. Pittsburgh and Amsterdam exhibit different urban morphologies—building heights, block dimensions, street-width distributions, and intersection densities all differ—so the depth-based *D* values optimized for SF cannot capture those geometries. The effect is most pronounced on AmsterTime, where the historical-modern matching task benefits from larger *D* values that enforce stronger viewpoint selectivity; the depth-aware *D* distribution (mean 7.5 m) falls below that preferred regime (optimal near *D* = 20 per Table 1).

This limitation is an instance of the general domain-shift problem in learning-based VPR and is not unique to our formulation. Importantly, the proposed framework is not inconsistent with cross-dataset deployment; rather, it requires depth statistics representative of the target region. We therefore view our method as a template that should be instantiated with depth information matched to the deployment domain. Three concrete future directions follow: (1) multi-city training using per-city depth caches merged before quantile mapping, so the resulting *D* distribution reflects geometric diversity across regions; (2) test-time *D* adaptation driven by a small, inexpensive depth sample from the deployment area; (3) a learnable *D*-selection head trained jointly with the descriptor extractor, conditioned on a coarse geometric signature (e.g., the per-cell SVD eigenvalue ratio), to produce transferable *D* estimates without external depth models at inference.

### 5.3. Practical Deployment Considerations

A central property of our method is that the depth-aware adaptation is confined entirely to the training pipeline. The deployed model is the unmodified ResNet-18 descriptor extractor: identical architecture, identical parameter count, and identical inference cost relative to baseline EigenPlaces. This matters for real-time deployment scenarios such as autonomous driving, where any added inference latency is measured against tight control loops. For large-scale on-vehicle deployment, depth preprocessing is executed once per training corpus—not per vehicle and not per query. A single map coverage campaign (for example, the 60k SF-XL panoramas) completes in approximately 2 h on a single RTX 3090 and produces a 28 KB per-cell *D* cache that is distributed with the trained model weights. Incremental map expansion follows the same pipeline and scales linearly with coverage area. Crucially, on-vehicle inference performs only descriptor extraction and nearest-neighbor retrieval, both of which are identical to baseline. For fleet-scale deployment, the preprocessing workload is parallelizable across GPUs and amortized across every vehicle using the map, making the additional training-time cost negligible against the operational benefit.

### 5.4. Relationship to MutualVPR

Our approach is complementary to, rather than competitive with, other adaptive VPR methods. MutualVPR [13] addresses a related but distinct problem: supervision inconsistencies caused by the rigid fixed-rule class construction of EigenPlaces. MutualVPR replaces the SVD-and-focal-point framework with an adaptive clustering mechanism that dynamically reassigns cell membership, solving label inconsistency at the cost of abandoning the geometrically interpretable focal-point formulation. Our method, conversely, retains the SVD-and-focal-point framework and improves its single weakest hyperparameter (the global scalar *D*) using an external geometric signal (per-cell monocular depth). The two directions therefore operate at orthogonal stages of the training-label pipeline: MutualVPR at the cluster-assignment stage, ours at the focal-geometry stage. A combined system—adaptive clustering with depth-aware per-cluster *D*—could, in principle, benefit from both, and we view this integration as a natural avenue for future work. More broadly, the body of adaptive VPR work (e.g., Refs. [10,16,17,18]) tends to focus on descriptor aggregation or on pretraining data scale; to our knowledge, our work is the first to make the training-label generation geometry itself adaptive, which places it in a distinct design space rather than in direct performance competition with any single prior method.

## 6. Conclusions

We presented a systematic analysis of the focal distance parameter *D* in EigenPlaces, revealing that optimal *D* values are strongly dataset-dependent, with performance gaps of up to 4.4 percentage points across benchmarks. Based on this analysis, we proposed a depth-aware adaptive strategy to replace the globally fixed *D* with per-cell values derived from monocular depth estimation. Our experiments demonstrated that (1) no single fixed *D* is universally optimal, validating the need for adaptive approaches; (2) the mean *D* value is the primary factor governing cross-dataset performance, with lower-mean strategies favoring same-distribution evaluation and higher-mean strategies benefiting cross-domain scenarios; and (3) depth-based adaptive signals outperform the SVD eigenvalue ratio proxy, confirming that scene geometry provides a more informative basis for *D* selection than coordinate statistics alone.

The proposed method requires no architectural changes and adds negligible training cost beyond a one-time depth estimation preprocessing step. Our analysis provides a practical framework for practitioners to select *D* values based on the relationship between training data geometry and deployment scenarios. Future work will explore multi-city depth-aware training, test-time *D* adaptation for deployment-specific optimization, and integration with larger backbone architectures to further improve cross-dataset generalization.

## Figures and Tables

**Table 1:** Recall@1 (%) for different fixed focal distance *D* values (groups_num = 1, 10 epochs). Bold indicates best per dataset.

*D* (m)	Pitts30k	AmsterTime	SF-XL val
0	86.2	27.4	87.7
5	87.5	29.8	**88.8**
10	87.3	31.6	86.5
20	**87.7**	**31.8**	85.0
30	87.6	31.5	84.7

**Table 2:** Multi-strategy ablation (groups_num = 1, 10 epochs). *D* μ and *D* σ denote the mean and standard deviation of assigned *D* values across cells.

Strategy	*D* μ	*D* σ	Pitts30k	AmsterTime	SF-XL val
Fixed *D* = 0	0	0	86.2	27.4	87.7
Fixed *D* = 5	5	0	87.5	29.8	88.8
Fixed *D* = 10	10	0	87.3	31.6	86.5
Fixed *D* = 20	20	0	87.7	31.8	85.0
Fixed *D* = 30	30	0	87.6	31.5	84.7
Depth raw	5.6	1.2	87.6	29.0	88.6
Depth scaled	15.3	3.3	87.8	31.5	85.4
SVD ratio	15.6	7.0	87.3	31.1	85.9
DQ [2,30]	16.0	8.1	87.4	32.3	86.4
DQ [3,12]	7.5	2.6	87.5	30.2	88.1
DQ [5,15]	10.0	2.9	87.3	31.3	87.1

**Table 3:** Main results (groups_num = 9, 40 epochs, ResNet-18/512). Bold indicates the best among our trained models.

Method	Pitts30k R@1	AmsterTime R@1	SF-XL val R@1
Baseline *D* = 10	**88.5**	**33.5**	88.3
Ours (DQ [3,12])	88.3	31.2	**88.9**
CosPlace [11] official	89.6	39.0	90.7
EigenPlaces [12] official	90.4	37.5	92.8

## Data Availability

The data presented in this study are available on request from the corresponding authors due to privacy restrictions.
